# Characteristics and research status among clinical trials in cardio‐oncology by bibliometric and visualized analysis

**DOI:** 10.1002/cam4.6045

**Published:** 2023-05-06

**Authors:** Liu Lini, Xu Rong, Huang Wei, Guo Xia, Xu Huayan, Xie Linjun, Zhang Hongding, Ju Gao, Lin Chao, Guo Yingkun

**Affiliations:** ^1^ Department of Radiology, Key Laboratory of Birth Defects and Related Diseases of Women and Children of Ministry of Education West China Second University Hospital, Sichuan University Chengdu China; ^2^ Department of Hematology West China Second University Hospital, Sichuan University Chengdu China

**Keywords:** bibliometrics, cardiotoxicity, CiteSpace, clinical trials, oncology, visual analysis

## Abstract

**Background:**

We aim to establish the characteristics of published cardio‐oncology research of clinical trials by bibliometric analysis and to talk about the prospects and difficulties facing the development of cardio‐oncology.

**Methods:**

Search of data related to clinical trials in cardiac oncology from 1990 to 2022 from the Web of Science core collection. Using CiteSpace to perform co‐citation analysis of authors, countries (regions) and institutions, journals and cited journals, cited authors and cited literature, and keywords.

**Results:**

Of the 607 clinical trial studies, the number of papers published per year has increased over time. The regions with the greatest influence were North America (especially the United States) and Europe. Multicenter research has always been the focus of cardio‐oncology research, but cross‐regional cooperation was still lacking. Myocardial toxicity caused by anthracyclines has received the earliest attention and has been studied for the longest time. Meanwhile, the efficacy and cardiotoxicity of new anticancer drugs always came into focus, but at a slow pace. Few studies on myocardial toxicity were related to the treatment of tumors except breast cancer. Risk factors, heart disease, adverse outcomes, follow‐up, and intervention protection were the major hotspots revealed by co‐citation cluster.

**Conclusions:**

There is great potential for the development of clinical trials in cardio‐oncology, especially in multicenter cooperation across different regions. Expansion of tumor types, myocardial toxicity of different drugs, and effective interventions in the research direction and design of clinical trials are necessary.

## 1 INTRODUCTION

With the continuous improvement of cancer treatment technology, patient survival times are improving and cancer has become increasingly a chronic disease.^1^, ^2^, ^3^ Moreover, the complications of cancer have become the main factor affecting the survival time and quality of life in patients,^4^, ^5^ especially the cardiovascular toxicity related to cancer treatment. Epidemiological studies reveal that cancer patients associated with cardiovascular diseases (CVDs) have a 3.78 times higher risk of all‐cause mortality than patients without CVDs, and CVDs killed 8.8% of cancer patients prematurely during long‐term survival.^6^ Some researchers have published reviews of the documentation on cardiotoxicity due to cancer treatment,^7^, ^8^, ^9^ and the incidence, clinical manifestations, monitoring, and/or protection were summarized. Besides, some authoritative organizations or institutions, such as the American Society of Clinical Oncology (ASCO), the Heart Failure Association (HFA), and the European Society of Cardiology (ESC),^10^, ^11^, ^12^, ^13^ have released clinical practice guidelines about cardio‐oncology. The imaging surveillance, treatment, or intervention recommendations are main challenges for oncologist that is lack of scientific evidence from randomized clinical trials. Clinical trials provide more powerful evidence for diagnosis and treatment which is important in cardio‐oncology. However, the development of clinical trials researches remains unclear and the summaries of cardio‐oncology are still lacking.

Bibliometric analysis is a mathematically and statistically based method for analyzing a large number of heterogeneous studies.^14^ The combination of visual processing tools, such as CiteSpace, helps to assemble data on a field's contribution from multiple perspectives, including different countries/regions, institutions, journals, authors, co‐citation networks, detailed research trends, or hotspots.^15^ It is a way to accurately capture and integrate data from different sources, visualizing the connections between complex data through knowledge maps. The knowledge maps obtained through CiteSpace allow readers to quickly know the major contributing countries, institutions and individuals and to have a clear understanding of the hot spots of the research direction.

The present study aims to provide a general understanding of the developments in clinical trials of cancer cardiotoxicity through a literature search and screening to analyze and summarize clinical trials of cardiotoxicity in oncology published since 1990. Interpreting and synthesizing these articles can help in forecasting possible trends and provide a reference for future researchers, especially those interested in but unfamiliar with the field.

## 2 MATERIAL AND METHODOLOGY

### 2.1 Database sources and strategies for searching

A bibliographic search was completed on April 28, 2022 to minimize bias due to database updates using the Web of Science Core Collection (WoSCC) database. The following search strategies were used: cardiotoxic* or cardiotoxicity (Topic) and cancer* or neoplasm* (Topic) and clinical trial (All Fields) and article (Document Type) and English (Language). The timespan was from 01‐01‐1990 to 28‐04‐2022 (publication date). After the preliminary data search, the two researchers individually filtered all the manuscripts to ensure their relevance to the topic of this study. Cases, papers that were not clinical trials, or papers that were reviews and meta‐analyses were excluded.

### 2.2 Bibliometric analysis

The search results were analyzed using Web of Science to extract histograms that showed the disciplinary distribution of publications. All records and citations for these publications were then transferred from the WoSCC database, preserved in .txt format and loaded into CiteSpace software V6.1.R2. Using the following configuration: time slices from January 1990 to April 2022, each slice 1 year. The selection used a modified g‐index in each slice: *g* ^2^ ≤ *k* ∑_*i*≤*g*_ *c*_*i*_, *k* ∈ Z^+^, *k* = 25. The procedure operated as a first step in data cleaning. If there were no replications, the original data were used directly; otherwise, the duplicates were removed prior to subsequent analysis. For coauthor network analysis, selecting one at a time for “Country,” “Institution,” and “Author” in the Node Type parameters section, leaving the rest of the settings as defaults. In the co‐citation analysis, once the data were imported into CiteSpace, nodes were selected as “Reference,” “Cited Journal,” and “Cited Author”, respectively. For keyword analysis, the relevant parameters were selected below: “Keyword” as the Node Type, the “Cosine” was chosen to calculate the strength of the relationship, and the pruning parameter regions “Pathfinder” and “pruning slicing network” were used to simplify the network and highlight the important structural features of the network. In the keyword burst detection, selecting “Keyword” as the node type and then using “Cosine” to calculate the burst intensity. Finding the top 40 keywords with the most explosive intensity. The results were presented as visual graphs in nodes, the more frequently the element appeared or was referenced, the larger the size of the node. The word similarities in the literature were categorized and scored by a specific algorithm, then the highest scoring word in each cluster was picked as the representative, the tag of the cluster. Full details were sorted out and displayed in Microsoft Word 2019.

## 3 RESULTS

### 3.1 Analysis of publications

**FIGURE 1:**
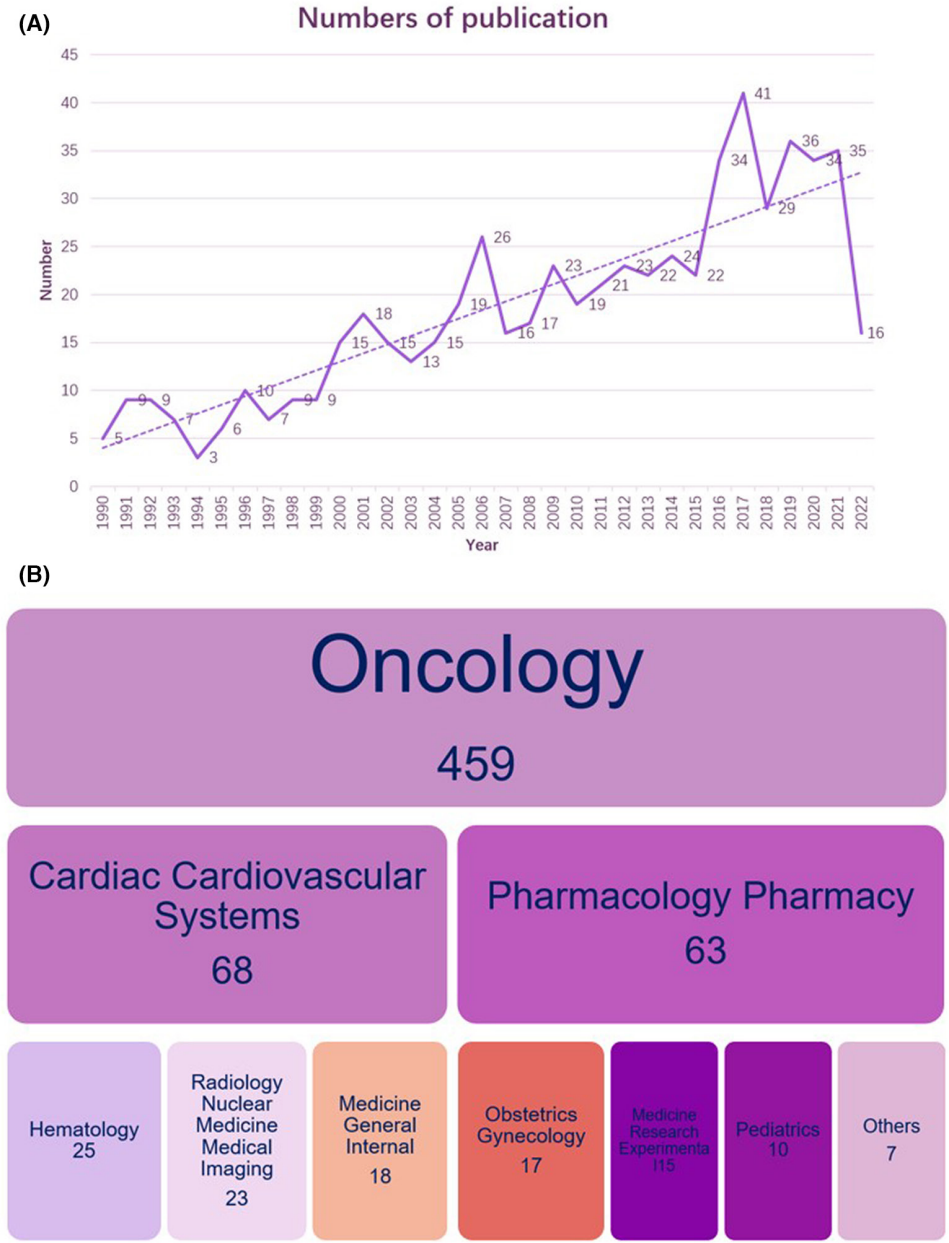
Number of publications per year and disciplinary distribution. (A) Annual quantitative distribution; (B) disciplinary distribution.

Altogether 953 of these publications matched the inclusiveness criteria under our search strategy. A total of 345 articles were excluded based on the exclusion criteria, covering 15 cases, 42 papers that were not clinical trials, and 288 papers that were reviews and meta‐analyses. After removing duplicate entries, 607 clinical trial papers were finally included in the analysis. The annual number of publications and disciplinary distribution of cardiotoxicity clinical trials in oncology are shown in Figure 1. The quantity of clinical trials published each year was limited to a certain number, but the overall trend was gradually increasing. Among the clinical studies on tumor cardiotoxicity, the most were published in oncology (75.6%, 459/607), and other included cardiac cardiovascular systems (11.2%, 68/607), pharmacology pharmacy (10.4%, 63/607), hematology (4.0%, 25/607), radiology, nuclear medicine and medical imaging (3.8%, 23/607), medicine general internal (3.0%, 18/607), obstetrics gynecology (2.8%, 17/607), medicine research experimental (2.5%, 15/607), and pediatrics (1.6%, 10/607).

### 3.2 Co‐authorship network analysis

**FIGURE 2:**
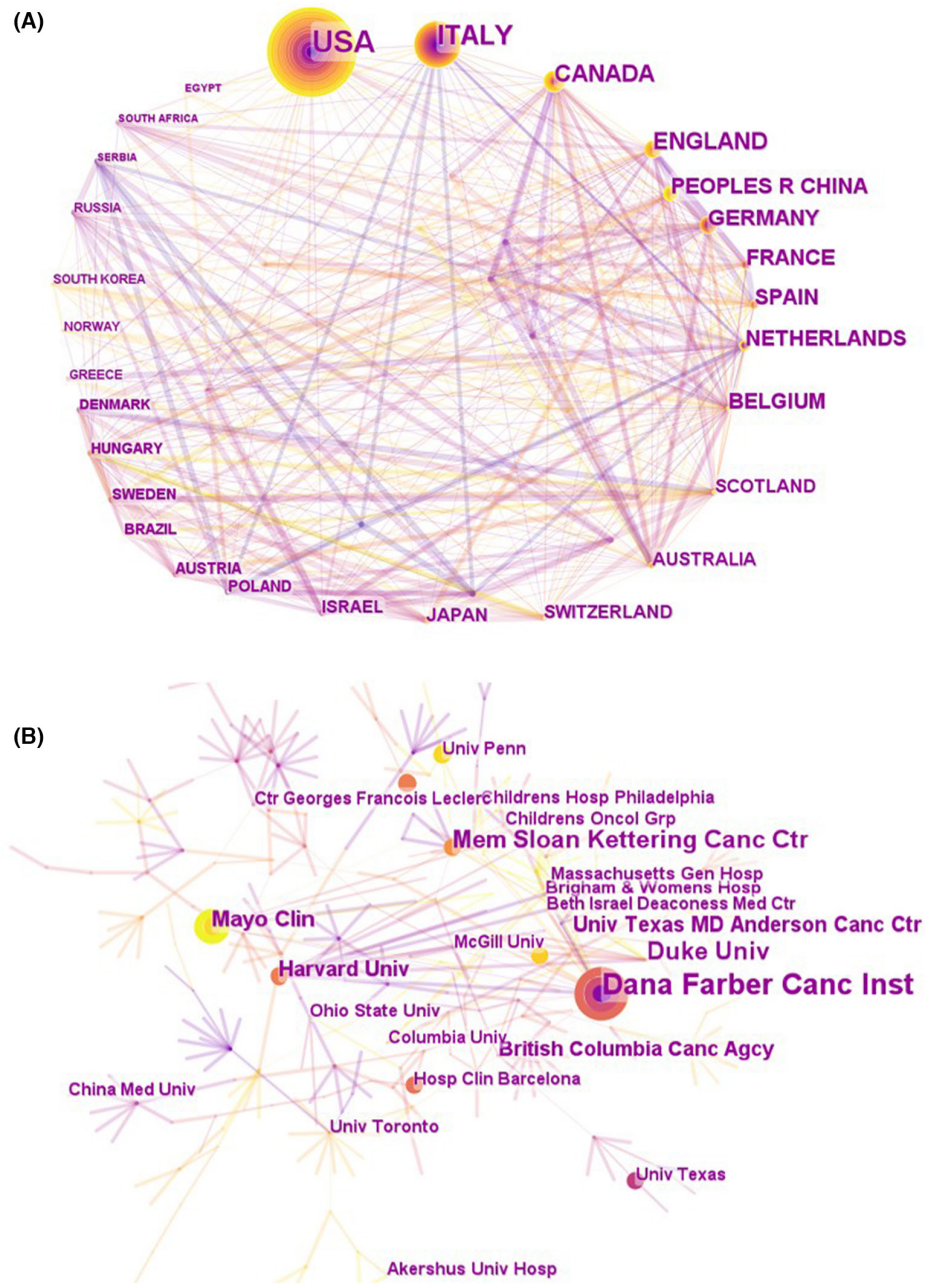
National networks and institutional networks of co‐authors for clinical trials of cardiotoxicity in oncology from 1990 to 2022 accessed via CiteSpace. (A) Collaborative country network visualization map (based on co‐author countries) revealed the impact of each node; (B) Visualization of co‐authored institutional networks by degrees of citation; countries and institutions with more than five publications can be seen in the map.

**FIGURE 3:**
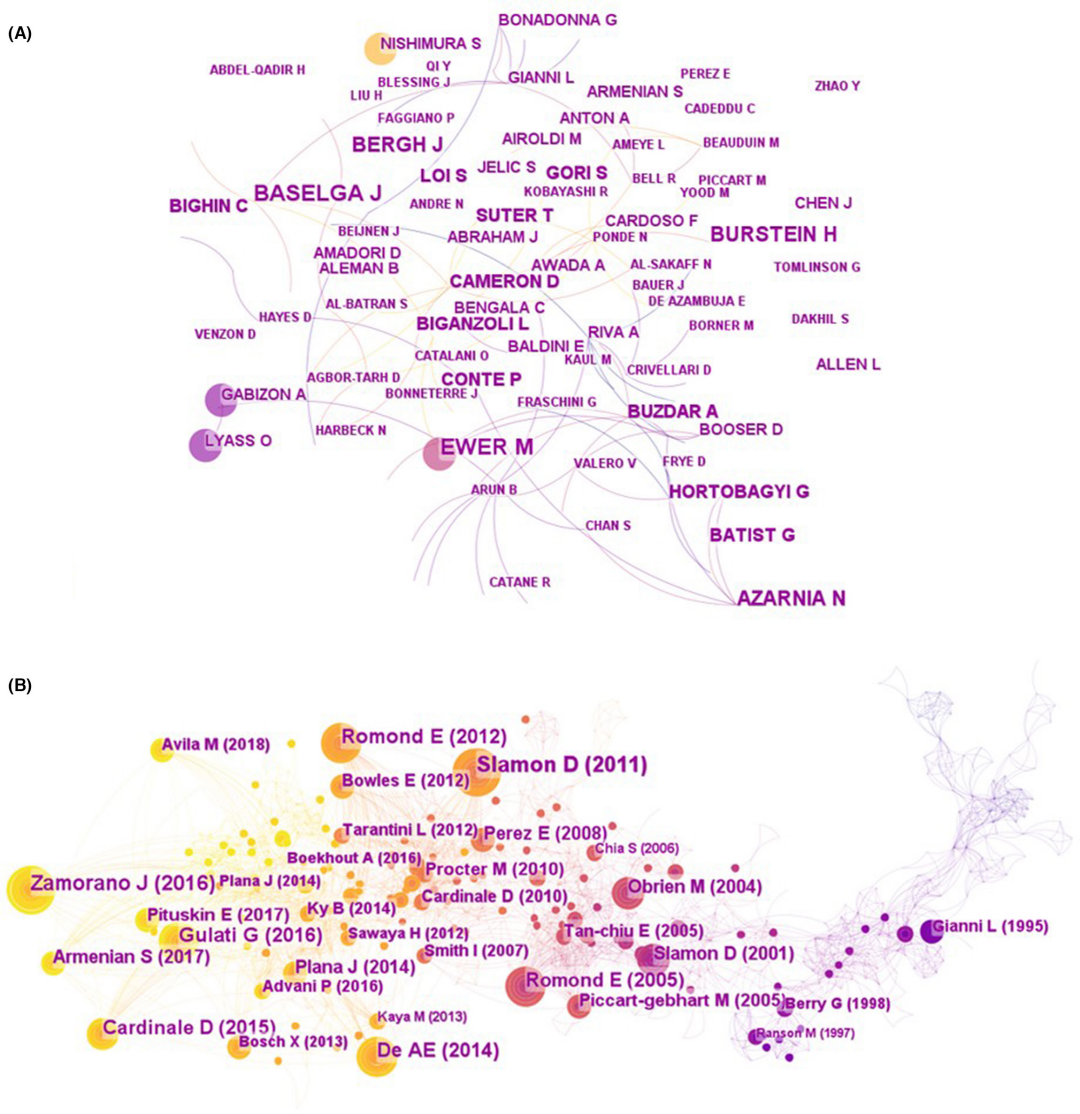
Networks of co‐authorship and literature co‐citations. (A) The links between each author represented collaboration (co‐authorship). Individual links between authors indicated collaborations (co‐authors). Node size as a proportion of number of co‐authors of the author. The figure showed author labels that had published more than two studies. (B) A network of reference co‐citations. Round nodes indicated citations; the line between the nodes suggested the frequency with which two references were cited simultaneously.

A country analysis of the distribution of these publications indicated that the 10 countries with the most collaborative papers were as follows: the United States (217, 35.7%), Italy (112, 18.5%), Canada (59, 9.7%), England (56, 9.2%), China (48, 7.9%), Germany (48, 7.9%), France (42, 6.9%), Spain (37, 6.1%), the Netherlands (37, 6.1%), and Belgium (34, 5.6%). Their relationship is displayed in Figure 2A. The co‐authorship between institutions is shown in Figure 2B. The Dana‐Farber Cancer Institute was the most published research institution, closely followed by Duke University and Mem Sloan Kettering Cancer Center. The details of the articles published by country and institution are shown in Table S1. There were many authors engaged in clinical studies of cancer cardiotoxicity, and Figure 3A shows authors who had published more than two studies. The top three authors were Thomas Suter, Santoro, and Azarnia.

A co‐citation was defined as being cited by two other texts at the same time.^16^ Co‐cited authors and co‐cited journals were then retrieved from the co‐cited documents. There was a total of 19,392 references in 607 articles, among which 1057 references were co‐cited references, with a total number of citations of 2417. The co‐cited references map shows the relationship between these documents (Figure 3B). The Journal of Clinical Oncology, New England Journal of Medicine, Circulation and Annual Oncology published most of top 10 in terms of total citations (Table 1). Furthermore, these journals were in the top ten co‐cited journals and the other six of the ten were Cancer, Lancet, European Journal of Cancer, British Journal of Cancer, Cancer Research and Seminars in Oncology. Slamon wrote the article that was cited most frequently, who along with Romond E and Cardinale D were the top three with the highest citations. Full details of the top 10 co‐cited journals and authors could be found in Table S2.

**TABLE 1:** Top 10 most cited articles.

Rank	Title	Year	Journal	First author	TC
1	Adjuvant trastuzumab in HER2‐positive breast cancer^17^	2011	N Engl J Med	Slamon D	31
2	2016 ESC Position paper on cancer treatments and cardiovascular toxicity developed under the auspices of the ESC Committee for Practice Guidelines: The Task Force for cancer treatments and cardiovascular toxicity of the European Society of Cardiology (ESC)^18^	2016	Eur Heart J	Zamorano JL	27
3	Seven‐year follow‐up assessment of cardiac function in NSABP B‐31, a randomized trial comparing doxorubicin and cyclophosphamide followed by paclitaxel (ACP) with ACP plus trastuzumab as adjuvant therapy for patients with node‐positive, human epidermal growth factor receptor 2‐positive breast cancer^19^	2012	J Clin Oncol	Romond EH	25
4	Prevention of cardiac dysfunction during adjuvant breast cancer therapy (PRA a 2 × 2 factorial, randomized, placebo‐controlled, double‐blind clinical trial of candesartan and metoprolol)^20^	2016	Eur Heart J	Gulati G	23
5	Trastuzumab‐associated cardiac events at 8 years of median follow‐up in the Herceptin Adjuvant trial (BIG 1–01)^21^	2014	J Clin Oncol	Azambuja E	23
6	Trastuzumab plus adjuvant chemotherapy for operable HER2‐positive breast cancer^22^	2005	N Engl J Med	Romond EH	22
7	Early detection of anthracycline cardiotoxicity and improvement with heart failure therapy^23^	2015	Circulation	Cardinale D	22
8	Reduced cardiotoxicity and comparable efficacy in a phase III trial of pegylated liposomal doxorubicin HCl (CAELYX/Doxil) versus conventional doxorubicin for first‐line treatment of metastatic breast cancer^24^	2004	Ann Oncol	O'Brien ME	19
9	Use of chemotherapy plus a monoclonal antibody against HER2 for metastatic breast cancer that overexpresses HER2^25^	2001	N Engl J Med	Slamon DJ	19
10	Cardiac safety analysis of doxorubicin and cyclophosphamide followed by paclitaxel with or without trastuzumab in the North Central Cancer Treatment Group N9831 adjuvant breast cancer trial^26^	2008	J Clin Oncol	Perez EA	18

### 3.3 Keyword analysis

**FIGURE 4:**
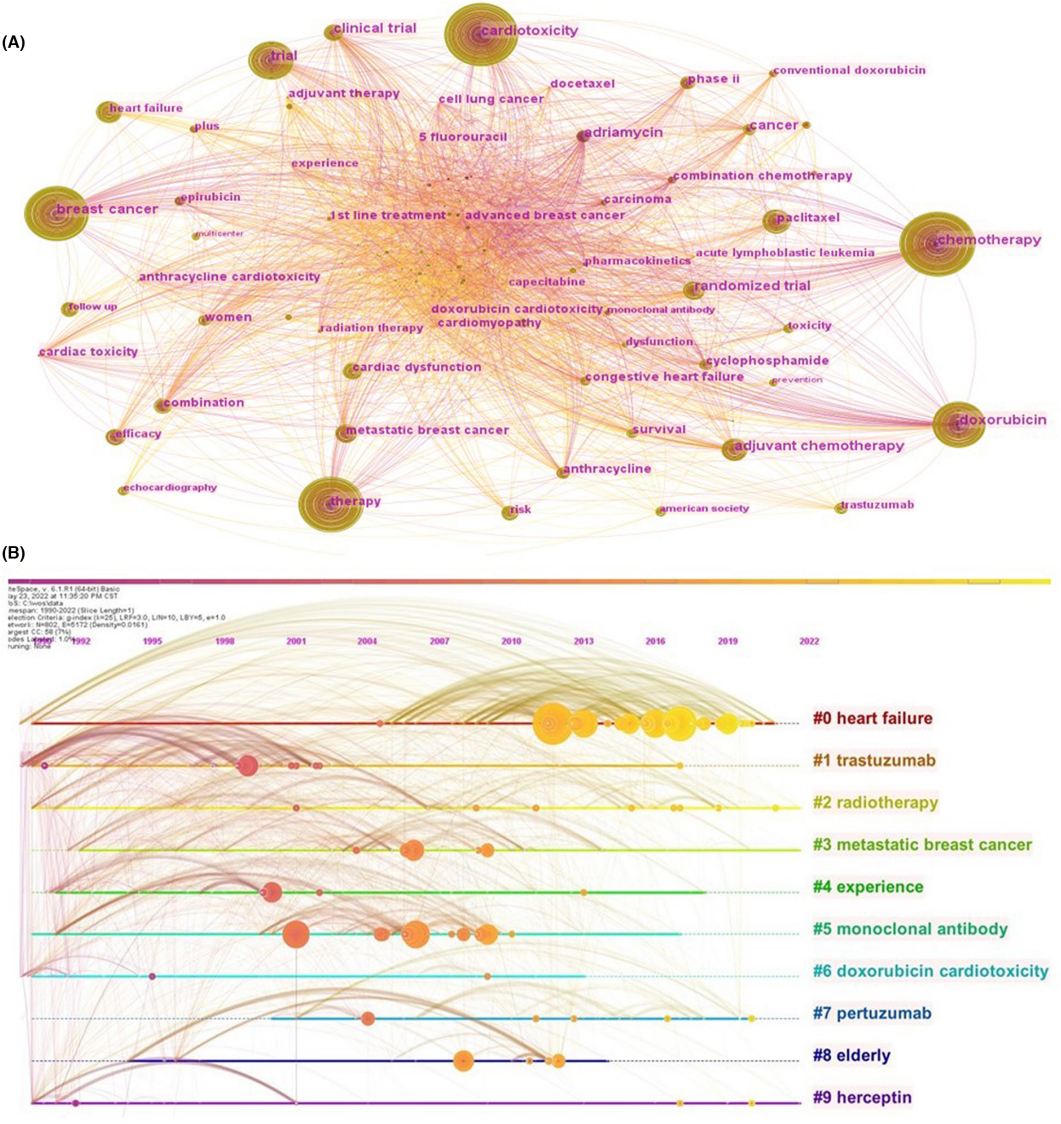
Keyword co‐occurrence and clustering. (A) Network of major keywords for publications about clinical trials of cardiotoxicity in oncology; (B) Chronological view of the top 10 most cited article clusters in the area of cardiotoxicity clinical trials in oncology.

**FIGURE 5:**
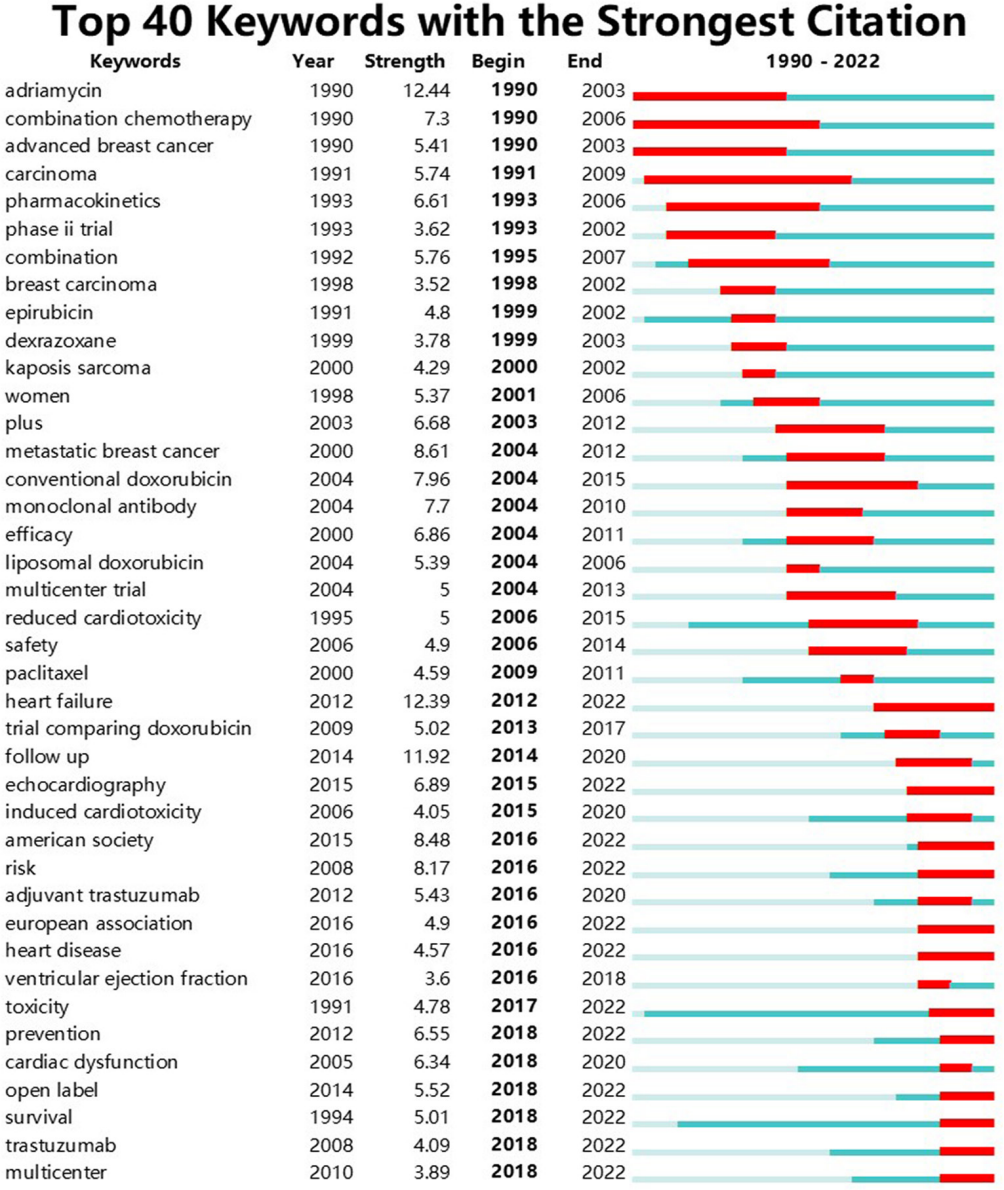
Keywords with the strongest article burst intensity on clinical trials of cardiotoxicity in oncology between 1990 and 2022. Keywords highlighted in red indicates a spike in the frequency of use of the keyword over the period. Blue represents a period of comparative decreasing popularity; Year means the first year when keyword was detected from our documents; Strength of each keyword was calculated by the software CiteSpace and the higher the number, the hotter the topic; Begin and end year represents the beginning and end of a popular research discipline.

As a highly condensed version of the content of the paper, to a certain extent, the keywords could summarize the topic of the paper in a simple and direct way. A keyword co‐occurrence network was an analysis method based on text content. The top 10 keywords used were as follows: cardiotoxicity, chemotherapy, breast cancer, therapy, doxorubicin, trial, paclitaxel, heart failure, randomized trial, and adjuvant chemotherapy. The circles in Figure 4A represent keywords and the larger the circle is, the more frequently the keyword appears. When the quantity of keywords was too large, it was rather difficult to identify the research topic to which they belong, and cluster analysis could be helpful in solving this problem (Figure 4B). Figure 4B shows the top 10 keyword clusters based on the log‐likelihood ratio (LLR) algorithm. They demonstrated many areas of concern for cancer cardiotoxicity research, including patients (#3 metastatic breast cancer and #8 elderly), treatment‐related (#1 trastuzumab, # 2 radiotherapy, #5 monoclonal antibody, #7 pertuzumab, and # 9 Herceptin), and complications (# 6 doxorubicin cardiotoxicity and # 0 heart failure). The burst detection results revealed some articles that have attracted the attention of fellow scientists. Citation bursts recorded the duration and intensity of each burst or the duration and intensity of the burst state, respectively. Application of keyword burst detection allowed quick insight into future research trends (Figure 5). A red line indicates a sudden increase in the use of a keyword during the relevant period. By contrast, a blue line illustrates relative unpopularity.

The keyword burst detection identified adriamycin as the top hot topic during 1990–2012. Moreover, heart failure and follow‐up have been the top two focal points from 2013 to 2022. Within the top 40 keywords with the strongest burst strength, chemotherapeutic drugs accounted for a large portion, including adriamycin, epirubicin, dexrazoxane, conventional doxorubicin, monoclonal antibody, liposomal doxorubicin, paclitaxel, trial comparing doxorubicin, adjuvant trastuzumab, and trastuzumab. Some diseases became the focus of the researchers several times before 2012, such as advanced breast cancer, carcinoma, breast carcinoma, Kaposis sarcoma, and metastatic breast cancer. But few specific diseases had become hot spots since 2012. Reduced cardiotoxicity, heart failure, induced cardiotoxicity, heart disease, ventricular ejection fraction, and cardiac dysfunction, which were closely related to heart disease, had gained the attention of scientists since 2006.

## 4 DISCUSSION

### 4.1 Generation information

We conducted a documentary bibliometric analysis of 608 cardiac oncology clinical trials in the WoSCC database from 1990 to 2022. This paper summarizes the cooperative relationship and research topics in the field of tumor cardiology, which provides value for future multi‐regional cooperation and clinical trials.

First, our literature analysis shows that this is a topic of great interest, with researchers from all over the world working in this direction. Since the starting point of our literature search, the number of clinical studies on tumor cardiotoxicity published each year has generally increased. Although it is increasing every year, the absolute quantity is not very large, which also reflects that there are certain difficulties in the implementation of research on this topic. In addition, a record 16 articles have been published to date in 2022, and while this count does not reflect the full‐year output, it is expected to increase in 2022.

Oncology is the discipline that published the most clinical studies in oncology cardiology, accounting for more than three‐quarters of the literature published in this field. The study of cancer disease itself remains the focus, with other disciplines such as cardiovascular systems and pharmacology reflecting a focus on symptoms and oncology drugs. The proportion of imaging medicine and nuclear medicine indicates that the detection method of cardiotoxicity of tumors has also received certain attention.

The co‐authors' map of countries and regions shows that the links between countries are strong, with the United States at the center of cooperation. Of the top 10 countries, only China is from Asia, and the others are European and American countries. Combined with the characteristics of ethnic distribution, more studies are needed in various countries to obtain the characteristics of tumor cardiology in Asian populations. In consistency with the nationwide dispersal, eight of the 10 (80%) most creative institutions are from the United States, with the remaining two belonging to the United Kingdom and Norway. In addition, four of the 10 institutions are cancer centers, four are general hospitals, and the remaining two are universities. Cancer centers and large hospitals have an advantage in conducting this kind of research because clinical research on tumor cardiology requires clinical patients.

For the co‐authorship map, there is very little collaboration between the authors, and their relationships are scattered. Santoro,^27^, ^28^ Thomas Suter,^29^, ^30^, ^31^, ^32^, ^33^ and Azarnia^34^, ^35^, ^36^ are the top three most productive authors. They are both experts in the field of oncology and have conducted multiple studies and published many high‐quality articles in their respective areas of expertise. However, they are not frequently cited by other authors. Slamon, from the Jonsson Comprehensive Cancer Center, University of California‐Los Angeles, is the author with the highest citations and published the top 1 cited article with the highest citations.^17^ Obviously, the author has made remarkable contributions to the chemotherapy treatment of breast cancer.

The most quoted articles in a given period may be a milestone in the development of a particular period. Among the top 10 cited articles, eight of the studies involved breast cancer patients,^17^, ^19^, ^20^, ^21^, ^22^, ^24^, ^25^, ^26^ and all were published in high‐quality magazines, which means that this research area has received significant attention and has made significant progress. An analysis of the top 10 favored journals demonstrates that 60% (6/10) of the journals have an impact factor greater than 10. These journals differ greatly in their impact factors, showing a polarization, which indicates that the research of tumor cardiology is full of opportunities and challenges. In summary, these papers are of great significance to the study of tumor cardiology.

### 4.2 Keywords

Keywords reflect on the central theme and essential content of the article and can be reasonable characteristics of topical research. Furthermore, keyword burst detection is recognized as an indication of cutting edge or emerging trends in research.

The analysis of the keyword map shows two research focuses in oncological cardiology: The study population and the etiology of cardiotoxicity. Breast cancer patients accounted for the majority of the study subjects^17^, ^19^, ^20^, ^21^, ^22^, ^24^, ^25^, ^26^, ^33^ and different diseases are made up of different populations. Most pediatric cancer patients are children with leukemia^37^, ^38^, ^39^, ^40^ meanwhile the elderly are mostly patients with advanced breast cancer metastatic breast cancer and hematological malignancy.^41^, ^42^, ^43^, ^44^, ^45^ This is consistent with the epidemiology of different malignancies and their treatment modalities.^1^ Breast cancer is the most prevalent in women and the treatment modalities of chemotherapy and radiotherapy (especially in the left breast) are potentially harmful to the heart. Leukemia is the most common type of cancer in children and is often treated with anthracyclines the cardiotoxicity of which has received widespread attention^46^, ^47^, ^48^, ^49^

Paclitaxel and radiotherapy are closely linked to cardiac toxicity. Paclitaxel is used in chemotherapy regimens for a variety of malignancies,^50^, ^51^, ^52^, ^53^, ^54^, ^55^ and radiotherapy for chest tumors also causes an increased risk of cardiotoxicity.^34^, ^35^, ^36^, ^56^, ^57^, ^58^, ^59^ Its efficacy and side effects naturally become a new focus of research when a drug is developed and put into use. The adjuvant therapy of trastuzumab in breast cancer patients positive for HER‐2 has attracted attention since the beginning of the century.^19^, ^22^, ^25^, ^29^, ^51^ The observation of the therapeutic effect of trastuzumab in breast cancer and the development of analogized trastuzumab have been a concern of scientists.^29^, ^30^, ^31^, ^32^, ^33^, ^60^ Furthermore, trastuzumab has shown good results in oncology treatment and is also used for other tumors besides breast cancer.^61^, ^62^, ^63^, ^64^, ^65^, ^66^ Monoclonal antibodies representing HER‐2‐targeted therapy are the focus of tumor cardiology research, and trastuzumab, pertuzumab and herceptin are the representative drugs. While anthracycline chemotherapy drugs represented by doxorubicin also occupy a considerable proportion in cardio‐oncology research.^67^, ^68^ As early as the 1990s, cardiotoxicity caused by doxorubicin has been widely studied by scientists.^69^ Cardiotoxicity caused by anthracyclines has been an ongoing concern of pediatrics.^46^, ^70^, ^71^ However, the research on reduction of cardiotoxicity is unsatisfactory, especially the use of dexrazoxane in children.

According to citation bursts analysis, the topic of tumor treatment prognosis has become a research hotspot since 2006, when safety and reduced cardiotoxicity became keywords with the strongest citations. The concentration of study has switched more from cancer treatment itself to changes in cardiac function caused by cancer treatment. In recent years, heart failure and follow‐up have become the focus of clinical research.^72^, ^73^, ^74^, ^75^ Follow‐up time varies considerably between clinical trials, ranging from 18 months to 10 years. Heart failure is noted by many clinical medical practitioners as a serious adverse cardiovascular event, but progression to heart failure is often irrevocable. The subclinical myocardial injury in early stage may be more important for improving long‐term prognosis, and some earlier changes need to be attended to, such as left ventricular dysfunction and myocardial injury. The monitoring of cancer cardiotoxicity has gradually progressed from the initial exploration of detection methods to the early assessment of cardiotoxicity,^76^, ^77^, ^78^ more clinical trials on subclinical cardiotoxicity testing are required. Echocardiography and ventricular ejection fraction are current research hotspots and will remain hot in the future.^79^ For the methods, ultrasound and MRI will play a greater role in the study of oncologic cardiology.

### 4.3 Strengths and limitations

As far as we know, it is the first bibliometric analysis to provide a description of milestones and trends in clinical trials in cardiac oncology. This analysis has the merit of objectivity, as it illustrates areas of development in the field and spotlights areas that have not been completely explored. Yet, there are some restrictions to this study. First, we only retrieved data from the WoSCC database and did not include other databases, such as PubMed, so some articles were inevitably omitted. Second, due to the requirements of CiteSpace, our analysis only covers articles in the WOS database with English keywords or abstracts. Finally, bibliometric research is a quantitative analysis of academic publications that can only be performed in cited and indexed journals but not in unindexed journals, papers, books, or government reports. In forthcoming research, we will use methodological evaluations to gain a deeper perspective on this topic.

## 5 CONCLUSION

In conclusion, the findings of this bibliometric study provide insights into trends in the progress of clinical trials in oncology cardiology over the past 30 years. There have been numerous clinical trials of oncologic cardiology in breast cancer patients, which have continued to gain traction and have been the focus of scientists' attention in recent years but have gradually shifted from the initial focus on treatment drugs to prognosis. Heart failure has become a major concern in recent years, as has follow‐up. Cardiac function testing will become a new direction for scientists to conduct clinical trials in tumor cardiology.

## AUTHOR CONTRIBUTIONS

**Liu Lini:** Conceptualization (lead); data curation (lead); formal analysis (lead); investigation (lead); methodology (lead); project administration (lead); resources (lead); software (lead); supervision (lead); validation (lead); visualization (lead); writing – original draft (lead); writing – review and editing (equal). **Xu Rong:** Conceptualization (equal); data curation (equal); data curation (equal); formal analysis (equal); formal analysis (equal); funding acquisition (equal); funding acquisition (equal); investigation (equal); investigation (equal); methodology (equal); methodology (equal); project administration (equal); project administration (equal); resources (equal); resources (equal); software (equal); software (equal); validation (equal); validation (equal); visualization (equal); visualization (equal); writing – original draft (equal); writing – original draft (equal); writing – review and editing (lead); writing – review and editing (lead). **Huang Wei:** Writing – review and editing (supporting). **Guo Xia:** Project administration (supporting). **Xu Huayan:** Conceptualization (supporting); project administration (supporting); writing – review and editing (supporting). **Xie Linjun:** Project administration (supporting); writing – review and editing (supporting). **Zhang Hongding:** Visualization (supporting); writing – review and editing (supporting). **Ju Gao:** Data curation (supporting); project administration (supporting); supervision (supporting). **Lin Chao:** Software (supporting); visualization (supporting). **Guo Yingkun:** Conceptualization (supporting); project administration (supporting); resources (supporting); writing – review and editing (supporting).

## FUNDING INFORMATION

This work was supported by the National Natural Science Foundation of China (81971586, 81771897, 82071874, 81901712, 81771887); Sichuan Science and Technology Program (2020YFS0050, 2020YJ0029, 2017TD0005, 21ZDYF1967); Fundamental Research Funds for the Central Universities (SCU2020D4132); Clinical Research Finding of the Chinese Society of Cardiovascular Disease (CSC) of 2019 (No. HFCSC2019B01).

## CONFLICT OF INTEREST STATEMENT

There are no conflicts of interest.

## Supporting information


Table S1–S2

## Data Availability

The data that support the findings of this study are available from the corresponding author upon reasonable request.
