# Supplementary material for: Characteristics and research status among clinical trials in cardio‐oncology by bibliometric and visualized analysis
Source: Cancer Med. 2023 May 6;12(11):12535–47. doi: 10.1002/cam4.6045 (PMC10278509; doi:10.1002/cam4.6045)
Supplement: Supplementary file 1 — Table S1–S2 [file CAM4-12-12535-s001.docx]

Table S1 Top 10 most productive countries and institutions

| Rank | Country(count) | Institution(count) |
| --- | --- | --- |
| 1 | USA (217) | Dana-Farber Cancer Institute (20) |
| 2 | Italy (112) | Duke University (12) |
| 3 | Canada (59) | Mem Sloan Kettering Cancer Center (12) |
| 4 | England (56) | Mayo Clinic (9) |
| 5 | China (48) | Harvard University (8) |
| 6 | Germany (48) | Univ Texas MD Anderson Cancer Center (8) |
| 7 | France (42) | British Columbia Cancer Agency (7) |
| 8 | Spain (37) | Akershus University Hospital (6) |
| 9 | Netherlands (37) | Brigham & Women’s Hospital (6) |
| 10 | Belgium (34) | Children’s Hospital Philadelphia (6) |

Table S2 Top 10 journals and authors with the highest citations

| Rank | Journals (IF_2021_) | citations | Authors | citations | |
| --- | --- | --- | --- | --- | --- |
| 1 | J Clin Oncol (44.544) | 554 | Slamon D | | 155 |
| 2 | New Engl J Med (91.245) | 405 | Romond E | | 115 |
| 3 | Ann Oncol (32.976) | 342 | Cardinale D | | 94 |
| 4 | Cancer (6.860) | 243 | Seidman A | | 93 |
| 5 | Lancet (79.321) | 203 | Ewer M | | 93 |
| 6 | Eur J Cancer (9.162) | 179 | Perez E | | 91 |
| 7 | Brit J Cancer (7.640) | 175 | Gianni L | | 80 |
| 8 | Cancer Res (12.701) | 169 | Piccart-Gebhart M | | 79 |
| 9 | Semin Oncol (5.385) | 159 | Vonhoff D | | 78 |
| 10 | J Clin Oncol (44.544) | 156 | Swain S | | 70 |
